# Correlation between Oxidative Stress and Transforming Growth Factor-Beta in Cancers

**DOI:** 10.3390/ijms222413181

**Published:** 2021-12-07

**Authors:** Jinwook Chung, Md Nazmul Huda, Yoonhwa Shin, Sunhee Han, Salima Akter, Insug Kang, Joohun Ha, Wonchae Choe, Tae Gyu Choi, Sung Soo Kim

**Affiliations:** 1 Biomedical Science Institute, Kyung Hee University, Seoul 02447, Korea; cck608@khu.ac.kr (J.C.); mnhuda@uams.edu (M.N.H.); jac03032@khu.ac.kr (Y.S.); sunheehan@khu.ac.kr (S.H.); iskang@khu.ac.kr (I.K.); hajh@khu.ac.kr (J.H.); wchoe@khu.ac.kr (W.C.); 2 Department of Biochemistry and Molecular Biology, School of Medicine, Kyung Hee University, Seoul 02447, Korea; salima_2015@buhs.ac.bd; 3 Department of Biochemistry and Molecular Biology, UAMS Winthrop P. Rockefeller Cancer Institute, University of Arkansas for Medical Sciences UAMS, Little Rock, AR 72205, USA; 4 Department of Biomedical Science, Graduate School, Kyung Hee University, Seoul 02447, Korea

**Keywords:** reactive oxygen species, transforming growth factor-beta, tumorigenesis, metastasis, cancer

## Abstract

The downregulation of reactive oxygen species (ROS) facilitates precancerous tumor development, even though increasing the level of ROS can promote metastasis. The transforming growth factor-beta (TGF-β) signaling pathway plays an anti-tumorigenic role in the initial stages of cancer development but a pro-tumorigenic role in later stages that fosters cancer metastasis. TGF-β can regulate the production of ROS unambiguously or downregulate antioxidant systems. ROS can influence TGF-β signaling by enhancing its expression and activation. Thus, TGF-β signaling and ROS might significantly coordinate cellular processes that cancer cells employ to expedite their malignancy. In cancer cells, interplay between oxidative stress and TGF-β is critical for tumorigenesis and cancer progression. Thus, both TGF-β and ROS can develop a robust relationship in cancer cells to augment their malignancy. This review focuses on the appropriate interpretation of this crosstalk between TGF-β and oxidative stress in cancer, exposing new potential approaches in cancer biology.

## 1. Introduction

Transforming growth factor-beta (TGF-β) has been claimed to play a biphasic role in cancer progression, behaving as a cancer suppressor in the early stages of carcinogenesis and exercising an oncogenic function in the later stages of cancer [1]. TGF-β initiates the epithelial-mesenchymal transition (EMT) of transformed cells, which influences a cancer aggression and metastasis and is repeatedly observed to be elevated in carcinoma cells [2,3].

In typical biological circumstances, reactive oxygen species (ROS) are continuously generated by ROS producers, whereas ROS-scavenging systems eradicate them to ensure redox homeostasis. Abnormal ROS production and/or antioxidant activity can lead to redox imbalances that promote cancer progression and are a characteristic of various cancer types [4,5]. ROS initiate several effects of TGF-β through tumorigenesis, as they participate in the control of downstream TGF-β signal transduction including that of Smads, MAPKs, and NF-*κ*B [6,7]. Moreover, TGF-β can control the level of ROS by increasing their production, as well as decreasing the activity of antioxidative/scavenging systems [7,8]. Additionally, elevated ROS levels may increase TGF-β expression and promote the release of TGF-β, making this growth factor bioavailable and effective [9].

ROS can cause cell damage at the macromolecular level and the destruction of nucleic acids, whereas TGF-β can cause senescence in the initial stages of epithelial tumorigenesis [1], partially by a mechanism involving ROS production. Thus, strong interactions between TGF-β and oxidative stress that lead to cancer progression can be observed. The objective of this review is to reveal the significance of TGF-β in cancer and its molecular relationship with the oxidative stress generated by ROS in cancer-cell metastasis.

## 2. Reactive Oxygen Species

### 2.1. Biology of ROS

Generally, ROS are continuously generated in the human body, although ROS-scavenging systems eliminate unnecessary ROS to preserve redox homeostasis. In the case of abnormal ROS production or the malfunction of antioxidant systems, redox imbalance can occur, which promotes the initiation and progression of several types of cancer [4,5]. ROS act as cell-signaling molecules for regular biological activities, but they can also promote the destruction of numerous cellular organelles and activities, eventually interrupting normal functioning [10].

**Figure 1:**
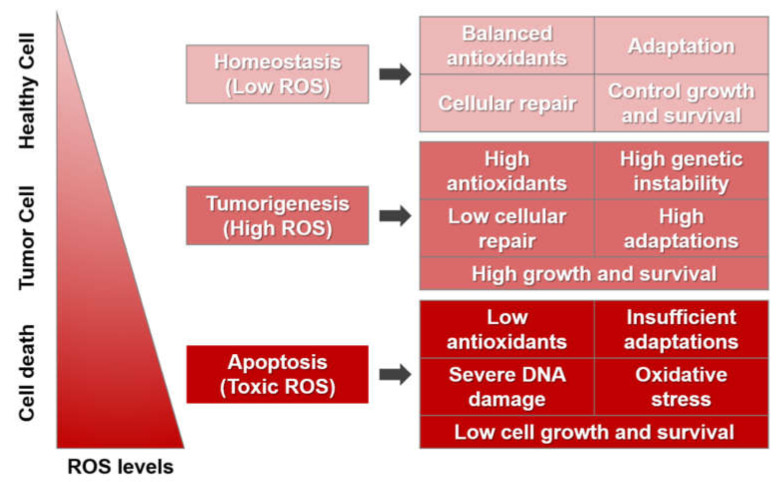
Model of different concentrations of ROS effects in cells.

Normal cells have sufficient adaptation power to defend themselves against the destructive effects of ROS. Low concentrations of ROS occur when there is adequate antioxidant activity for cellular repair and, therefore, limited cancer-cell survival and proliferation. The metabolic activity of cancer cells produces high ROS concentrations, increasing cell survival and proliferation, directing DNA damage, reducing cellular repair by constant DNA-damage-repair pathways, and promoting genetic instability. Higher ROS levels can cause cellular damage, though cancer cells adapt to stressful situations including hypoxia. Cancer cells show enhanced antioxidant activity, which eliminates excessive ROS while pro-tumorigenic signaling is sustained. If ROS rise significantly to toxic ROS concentrations, for instance, in response to chemotherapy-type ROS-generating agents, the oxidative stress can result in irreversible damage to the cell, insufficient adaptations, and, ultimately, the death of the cancer cell (Figure 1).

### 2.2. Roles of ROS in Cancer

In normal cells, the production and elimination of ROS are controlled and in equilibrium. By contrast, dysregulated oxidative stress in cancer cells might precede the chemical destruction of protein, lipids, and DNA, promoting damage to and mutations in cells that lead to tumorigenesis [11,12]. Several types of tumor growth and development are promoted by ROS depending on the mutagenic capability during tumorigenesis. Increased NADPH oxidase (Nox) activity, which correlates with ROS imbalance, leads to deregulated cellular receptor signaling, amplified metabolic activity, peroxisome activity, oncogene initiation, cyclooxygenase (COX) activation, lipoxygenase (LOX) activation, thymidine phosphorylase activation, and mitochondrial dysfunction [13,14]. The Nox/dual oxidase (Duox) family’s best-characterized member is Nox (Phox/Nox2), which increases the production of superoxide anion. The primary molecular mediator of Nox1, -2, and -3 is the small GTPase Rac1, whereas Rac1 does not significantly affect Nox4, Nox5, or Duox [13,15,16].

Abnormal or excessive activity of the antioxidant defense system generates cancer cells that are more resistant to neutralizing oxidative stress, which is also correlated with cancer treatment, including chemotherapy [17]. Several antioxidant systems show increased activity associated with the detoxification of ROS in cancer cells, which makes the cells more resistant to chemotherapy-induced toxicity [12].

This redox imbalance resulting from anomalous ROS production and deregulated antioxidant activity promotes tumor development and other diseases, including fibrosis [18,19]. Advanced disruption of the antioxidant cellular systems is accompanied by various tumor-associated metabolic effects related to redox imbalance, including uncontrolled cellular-receptor activity, disrupted peroxisomal and mitochondrial function, the overexpression of oncogenes, and the initiation of lipoxygenase phosphorylation, cyclooxygenase activity, and thymidine phosphorylase activity [12]. The wide-ranging effects of ROS lead to an abnormally high rate of proliferation of tumor cells. However, a high concentration of ROS can be cytotoxic as well as pro-tumorigenic. Thus, cancer cells can adapt to high ROS levels by evading the apoptosis elicited by ROS [20,21]. It is said that, in cancer, ROS show a dual character. Through angiogenesis and the NF-kB, HIF-1a, and EMT pathways, ROS can play a role in the initiation and maintenance of different signaling pathways participating in the proliferation of cells as a secondary messenger [22].

NADPH oxidase (Nox)-mediated ROS can regulate numerous metabolic actions, including cell migration and proliferation, gene expression, and angiogenesis [23]. The Nox-initiated overproduction of ROS leads to cancer initiation and development via oxidative stress. Among the several Nox isoforms, deregulated Nox2 and -4 are associated with cancer. In the case of renal and pancreatic cancers and invasive breast cancers, the overexpression of Nox4 has been demonstrated [24]. Cancer risk is correlated with the expression of different antioxidant enzymes such as glutathione peroxidase, glutathione-S-transferase, and glutathione reductase, and molecules such as glutathione (GSH) [11,25]. Metal-ion-dependent superoxide dismutase (SOD) is one of the main enzymes that participates in the antioxidant cellular defense, which defends cells from oxidizing toxic products generated during aerobic respiration. Brain-tumor patients show reduced SOD activity compared to normal patients, while breast and laryngeal carcinomas show elevated levels of SOD [26]. Additionally, invasive carcinomas express notably lower SOD than superficial carcinomas.

Glutathione (GSH) is the most crucial cellular non-enzymatic antioxidant, which is indispensable in numerous cellular antioxidant systems. GSH oxidation–reduction is an important intracellular pathway for ROS detoxification in normal cells. Lower GSH is related to low resistance against oxidative stress at the cellular level. More than a few types of cancers including breast, ovarian, lung, and head and neck cancers show elevated GSH compared to normal cells [27]. Numerous previous studies have shown that increased GSH levels are critical for both cancer initiation and proliferation [28], and the association of GSH with cancer is exploited for developing chemotherapy [29].

ROS can alter several cellular pathways; they can modify the DNA-binding sites of redox-sensitive transcription factors such as NF-ĸB, HIF-1α, AP-1, and p53 and the oxidation of the cysteine residues [30]. Multiple types of amino acids can also be oxidized by ROS, [31] leading to structural and conformational changes in tertiary proteins, which, in turn, initiate protein degradation. In addition, extracellular/intracellular ROS can cause DNA damage, triggering various stress-response genes and DNA-repair mechanisms. For example, the active transcription factor p53, a redox-sensitive protein, can initiate apoptosis, cell-cycle arrest, and senescence [32]; p53 performs a vital role by inhibiting DNA damage under excess ROS [33].

The requirements for nutrients and oxygen are enhanced while a primary cancer continues to develop, and a rapidly expanding cancer results in poor oxygen availability, as these requirements are not regularly met. Therefore, cancer cells undergo some alterations such as p53 mutation and HIF-1 activation to adapt to this oxygen- and nutrient-deprived situation [34]. In addition, a hypoxic microenvironment leads to ROS formation via the release of superoxide, hydrogen peroxide, and hydroxyl radicals from the electron transport chain of the mitochondria. ROS also promote the release of HIF-1α in normoxic as well as hypoxic conditions [35,36].

ROS can trigger various metastatic pathways such as the activity of matrix metalloproteinases (MMPs), which degrade basement membranes and extracellular matrices, leading to the intravasation and extravasation of tumor cells [37]. In addition, Nox-family-initiated ROS are vital for the development of actin-rich membrane protrusions in tumor cells [38]. In cancer cells, a decrease in the amount of ROS, mediated by antioxidants, can reduce cell viability, invasion, and actin-rich membrane protrusions, which indicates the function of antioxidants in alleviating metastasis [38,39].

Oxidative stress is extremely deregulated in cancer cells. Excessive ROS production leads to the oxidative damage of cellular compartments such as proteins, lipids, and particularly DNA, which promotes tumorigenic mutations. Meanwhile, abnormally highly activated antioxidant defense systems render cancer cells more resistant to oxidative stress and, thus, are involved in chemotherapeutic resistance [40].

## 3. Transforming Growth Factor-Beta

### 3.1. Transforming Growth Factor-Beta Signaling

**Figure 2:**
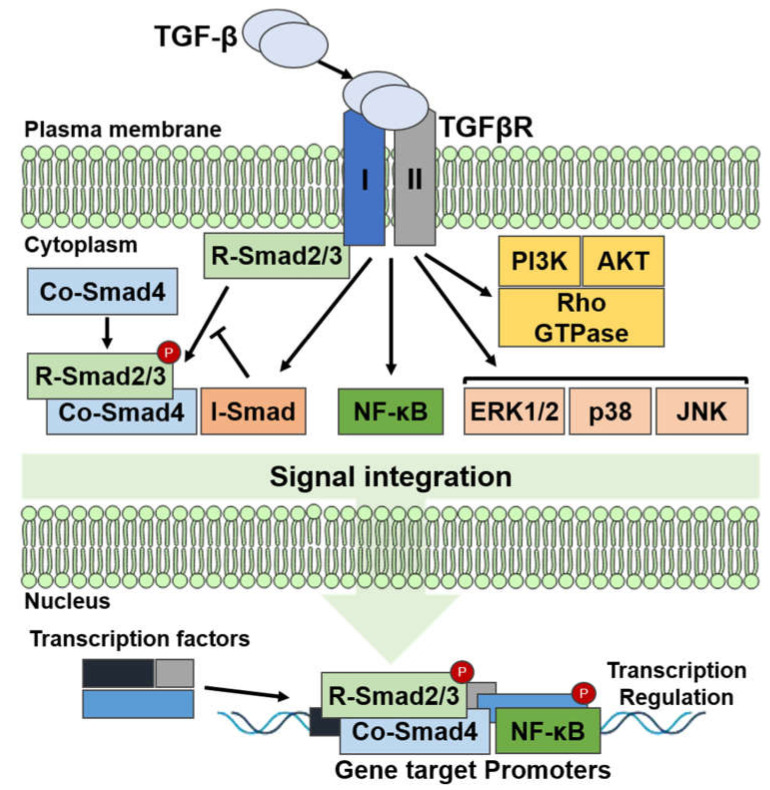
TGF-β signaling pathway.

Although transforming growth factor-beta (TGF-β) is involved in normal cellular development, including cell growth and differentiation, it can induce tumorigenesis. In the case of TGF-β signaling, a TGF-β dimer binds to the TGF-β type I receptor (TβRI/ALK5) and the TGF-β type II receptor (TβRII). TβRI/ALK5 is activated and phosphorylated by the TGF-β dimer and TβRII complex. Then, the activated type I receptor phosphorylates the cytoplasmic mediators Smad2 and -3, which accelerate the discharge of Smads, and then, the activated Smad2 and -3 attach to the common Smad4 (co-Smad4) and move to the nucleus. The Smad complex switches the expression of various target genes by intermingling with several repressors, transcription factors, or coactivators [41,42,43] (Figure 2).

Inhibitory Smad proteins (I-Smads), i.e., Smad6 and Smad7, control TGF-β signaling. Smad7 interacts with TβRI and binds to and degrades TGF-β, whereas Smad6 forms a complex by binding with Smad1 and inhibiting BMP signaling [44]. Conversely, the expression of I-Smads is controlled by TGF-β signaling. A type III non-kinase receptor (TβRIII) that can establish a complex with further TGF-β receptors can control TGF-β’s activity [45]. Moreover, TGF-β signaling is controlled by post-translational modifications such as the phosphorylation, ubiquitination, and SUMOylation of the TGF-β/TGF-β receptor/Smad cascades, which, in turn, control the stability and accessibility of I-Smads. Several non-Smad pathways, such as the mitogen-activated protein kinases (MAPKs), phosphoinositide 3-kinase (PI3K), nuclear factor *κ*B (NF-*κ*B), RAC-alpha serine/threonine-protein kinases (AKT1 and 2), and cyclooxygenase 2 pathways are also triggered by TGF-β [46,47] (Figure 2).

### 3.2. Roles of Transforming Growth Factor-Beta in Cancer

TGF-β has dual properties, as it can function as a cancer suppressor and cancer promoter depending on the stage of cancer. In the early stage of cancer, TGF-β defends the injured or stressed epithelium from exposure to mitogens. Through alterations in the mechanisms of TGF-β signaling, for instance, disabling mutations in TβRII and Smad4, cancer cells develop tolerance to the tumor-suppressive effects of TGF-β in advanced stages of cancer [13,14,42].

TGF-β can act as an immunosuppressive cytokine facilitating the evasion of immune system surveillance, as well as stimulating the growth and metastasis of a tumor [48], thus functioning as an oncogene. Cancer cells produce higher amounts of TGF-β to stifle antitumor immune responses and establish an immunotolerant environment [49,50]. TGF-β can function as a chemoattractant for monocytes and macrophages, drawing them to tumor sites. Numerous cancers express elevated levels of TGF-β, which are associated with tumor development. Several cancer types show high levels of TGF-β in the plasma, which are linked to advanced stages of cancer, metastases, and inadequate clinical effects in breast cancer [51]. Higher levels of TGF-β in the plasma are observed in a number of cancer types such as prostate and pancreatic cancers, as well as myeloma patients, non-Hodgkin’s lymphoma, etc. [51,52,53]. At the time of tumor progression, the TGF-β response varies by carcinogenesis stage and the sensitivity of the cancer cells.

TGF-β is an essential cytokine in cancer cells with a role in tumorigenesis and cancer development depending on the cancer stage [54]. TGF-β triggers downstream signaling by the phosphorylation of different mediators, including Smad proteins that are vital effector molecules in the TGF-β signaling pathway [55]. Furthermore, the expression of specific target genes and the control of nuclear or cytoplasmic proteins are regulated by interactions involving Smad and related downstream proteins. Elevated levels of TGF-β are observed in numerous types of cancer, contributing to the cancer’s resistance and the ineffectiveness of clinical treatments [56]. The Smad4 gene appears to have a crucial function in carcinogenesis, especially in pancreatic, colorectal, and gastrointestinal cancers [57,58,59,60]. Ultimately, TGF-β allows cancer cells to evade immune surveillance and, thereby, promotes tumor growth and metastasis [59].

## 4. Relationship of TGF-β and ROS

### 4.1. TGF-β Regulates ROS Activity

**Figure 3:**
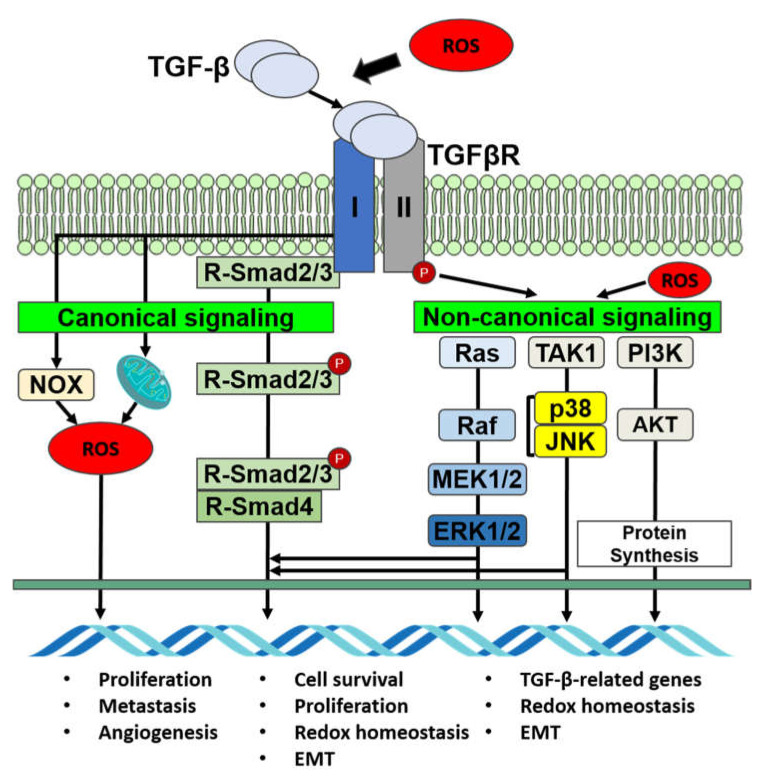
Signaling of TGF-β and oxidative stress in cancer cells.

TGF-β stimulates ROS production in different cellular compartments, mitochondria, and microsomes in hepatocytes, mink lung epithelial cells (Mv1Lu), and transformed and non-transformed cells [6,61,62,63]. The control of ROS and gene expression by TGF-β may be mediated by a cysteine thiol–disulfide swap reaction in the mitochondria [8]. TGF-β triggers Noxs in a Rac1-dependent manner, as well as Nox4 in different cells in vivo and in vitro [6,64]. Smad3 controls TGF-β to stimulate Nox4 gene expression, whereas in breast cancer cells, it is inhibited by wild-type p53 [65,66]. In pancreatic cancer, Nox4 is one of the primary sources of ROS, as TGF-β stimulates Nox4 gene expression together with a ROS increase, even though a decrease in Nox4 lowers ROS synthesis [67]. In adenocarcinoma Hela cells, TGF-β can, likewise, stimulate Nox2 gene expression. Moreover, TGF-β can decrease the expression of antioxidants such as glutaredoxin, catalase, glutathione peroxidase (GPx), superoxide dismutase (SOD), and glutathione (GSH), and thereby controls ROS activity [6]. The mitochondria are the major supplier of ROS in cells, whereas TGF-β enhances mitochondrial ROS production in various cell types. TGF-β can produce mitochondrial ROS through complex IV downregulation, which promotes the cell-cycle arrest of lung epithelial cells and apoptosis [68]. In normal human lung fibroblasts, TGF-β-mediated ROS production necessitates mitochondrial complex III activity, α-smooth muscle actin (α-SMA) and connective tissue growth factor (CTGF) [69]. In breast cancers, Smad3 controls Nox4 [65], and in pancreatic cancers, Nox4 delivers ROS for the EMT phenotype switch [67] (Figure 3).

### 4.2. TGF-β Signaling Is Mediated by ROS

Smad and non-Smad mechanisms are related to the activation of several signaling pathways by TGF-β. Smad2 signaling is influenced by ROS, as *N*-acetyl cysteine and lowered glutathione along with L-cysteine might prevent TGF-β-stimulated Smad2 phosphorylation [70], which suggests that thiol groups are significant for inhibiting Smad2 stimulation and the buildup of Smad2/Smad4 complexes in the nucleus [70,71]. In human skin fibroblasts, prolonged exposure to H_2_O_2_ prompts a decrease in TβRII and Smad3 expression, which weakens TGF-β signaling [72]. ROS can regulate the initiation of MAPKs facilitated by TGF-β through the activity of phosphatases that dephosphorylate tyrosine, serine, and threonine groups. TGF-β also initiates NF-*κ*B with the help of ROS [64]. The ROS-generated oxidation of NF-*κ*B impedes its DNA-binding capability, prompting destructive control at the nucleus compartment and the inhibition of NF-*κ*B’s transcriptional activities [73]. ROS can increase the expression and secretion of TGF-β and can work as a facilitator in the canonical and noncanonical pathways along with activating TGF-β from its latent form. It can initiate the overexpression of the TGF-β gene in various cells, including cultured A549 human epithelial cells, articular chondrocytes, human keratinocytes, human hepatocellular carcinoma, etc. [70,74,75,76] (Figure 3).

### 4.3. Association of ROS with the Activation of Latent TGF-β

TGF-β is initially synthesized as an inactive multiprotein precursor complex containing a latency-associated peptide domain, as well as mature TGF-β. The TGF-β sensors and particular types of oxidative stress may lead to the production of TGF-β during inflammation and apoptosis, which initiate extracellular matrix (ECM) damage through ROS production [11], promoting tumor progression. Interestingly, in cancer cells, oxidative stress/ROS and TGF-β establish a regulatory loop. TGF-β controls oxidative stress by both increasing ROS production and controlling the antioxidative system, whereas ROS controls Smad signaling, promoting the resistance of the cancer cells to the TGF-β-mediated inhibition of proliferation [77] in the initial stage of cancer, conflicting with the upregulation of the MAPK and NF-*κ*B pathways. Amplified ROS may enhance genomic mutation rates during cancer initiation [78]. Therefore, in the early stage of tumor progression, ROS can change the antitumorigenic function of TGF-β to a pro-tumorigenic function, as well as this ROS–TGF-β relationship being able to produce the cancer phenotype.

### 4.4. TGF-β and ROS Share Downstream Mediators

Phosphorylated Smad2/3 proteins facilitate the TGF-β signaling pathway through transcriptional regulators together with Smad4. Although TGF-β receptors can also facilitate Ras, MAPKs, NF-κB, and PI3K, which are non-Smad-mediated pathways, in pancreatic cells, TGF-β delays ERK activation [79], though it can also activate RAS–RAF–MEK–ERK signaling with ERK phosphorylation, which is analogous to the signaling of mitogenic factors such as epidermal growth factor-generated ERK activation [80]. ERK, in turn, controls target gene transcription through downstream transcription factors and Smads to regulate EMT. In addition, ROS can initiate MAPK pathways and facilitate NF-κB’s transcriptional activity, revealing that both ROS and TGF-β receptors cooperate through downstream factors among pathways [81]. The increase in ROS causes genetic instability, which may induce tumorigenesis (Figure 3).

## 5. Crosstalk of TGF-β and ROS in Cancers

### 5.1. Roles of TGF-β and ROS in EMT

Since TGF-β contributes to several differentiation processes involved in EMT, it is regarded a dominant regulator of EMT. Cancer cells repeatedly subjected to TGF-β elicit EMT, which performs a crucial role in cancer progression [82]. TGF-β can provoke EMT through Smad3 signaling, which, along with Smad4, is essential for EMT promotion [47,83,84,85]. In contrast to the function of Smad3, Smad2 acts as an inhibitor of EMT, as Smad2 ablation boosts EMT during skin carcinogenesis [86]. By contrast, Smad2 can contribute to TGF-β1-induced EMT, and the overexpression of constitutively active Smad2 increases EMT in cancer cells [87]. TGF-β enhances the production of extracellular matrix proteinases, augmenting tumor progression and initiation [42]. TGF-β increases the expression of the Nox4 gene, which contributes to ROS production through a Smad3-dependent mechanism, and it might be significant for the development of TGF-β-generated EMT in breast cancer [63]. The TGF-β-induced ROS-dependent or independent production of Smad3 may itself promote TGF-β expression [6,88] which might be responsible for the progress of EMT via a positive ROS–TGF-β feedback loop in cancer cells.

### 5.2. Roles of ROS and TGF-β in Cellular Senescence and Cancer

Elevated levels of ROS can damage proteins, lipids, and nucleic acids; promote cellular senescence; and promote the development of various age-associated diseases [89]. ROS might provoke genomic alterations and inhibit cancer-suppressor genes in addition to stimulating the expression of oncogenes. Additionally, ROS might reversibly control MAPK, PI3K, PKC, and phosphatases that are intracellular signaling targets for the development of cell malignancy. TGF-β dominates the early stages of epithelial tumorigenesis, cell-cycle arrest, apoptosis, genomic stability, and cellular senescence [1]. TGF-β hinders the activity of ANT2 in cancer cells by establishing an NF1/Smad4 complex, and this suppression of ANT2 promotes senescence-associated oxidative stress, as well as DNA damage [90]. Fibroblast-dependent TGF-β initiation is induced by the elevated levels of ROS in cancer, and this causes fibroblast senescence. Likewise, senescent fibroblasts have an elevated capability to promote malignant keratinocyte invasion in vitro [91].

### 5.3. Interplay within ROS and TGF-β Signaling in Cancer

In cancer cells, TGF-β participates in suppressing immune cells and fostering EMT and angiogenesis [92]. In immune cells, apoptosis is stimulated by the prompt production of cytolytic factors in T cells. It also prevents the multiplication and differentiation of various immune cells and reduces the tumor surface’s immunogenicity [93]. Thus, the obstruction of TGF-β signaling by T cells might improve antitumor immunity by producing CD8-mediated tumor-specific cytotoxic T-cell responses [94]. In addition, the Smad-dependent TGF-β signaling pathway might encourage vascular endothelial growth factor expression, which promotes angiogenesis, whereas different levels of TGF-β expression exhibit divergent impacts on angiogenesis [95].

### 5.4. TGF-β and Oxidative Stress Crosstalk in Cancer-Cell Metabolism

TGF-β can regulate the level of ROS by either increasing its production or by inhibiting antioxidant activity or cellular scavenging systems [6,8]. Furthermore, an increase in ROS can sequentially promote TGF-β expression and its release for activity [6,9]. Simultaneously, ROS can stimulate several TGF-β-utilized mediators that can regulate ROS levels by boosting their production and decreasing antioxidant activity.

ROS can facilitate several outcomes of TGF-β in cancer development by derestricting cancer suppressors and by boosting tumorigenesis. ROS are completely engaged in the control of the TGF-β pathway by involving several factors such as Smad, MAPK, and NF-kB, which act as promoters of cell proliferation and cell motility [7]. This is primarily realized by boosting Smad to make cancer cells tolerant to the inhibition of proliferation usually induced by TGF-β in the early stage of tumorigenesis [77]. Additionally, in cancer cells, TGF-β is engaged in several additional signaling pathways such as the I3K/Akt or MAPK pathways that control NF-kB, HIF-1a, or other redox-sensitive transcription factors [96].

The Nox family plays an essential role in facilitating the activity of TGF-β through the production of ROS that can be released in tumors. Nox4-generated ROS facilitate apoptosis, which, subsequently, is a TGF-β-mediated signaling pathway [97]. Nox4 can mediate TGF-β’s effects, while Nox-dependent redox signaling is capable of mediating TGF-β signaling. TGF-β can activate the expression of Nox2 gene as well as its protein’s activity [98]. TGF-β can reduce the concentration of GSH by preventing the expression of the catalytic subunit of the gamma-glutamylcysteine synthetase (GLC) enzyme [99]. From improving the invasiveness in tumor cells, the abnormal function of the mitochondrial complex III during hypoxic stress produces ROS, as well as HIF-1a stabilizing ROS, thus interacting with Snail throughout EMT [7,100].

The regulation of cellular redox homeostasis is associated with EMT, and oxidative stress significantly promotes cancer malignancy [14]. Oxidative stress can sequentially adjust the cellular response to TGF-β, progressively directing cancer cells to be destructive and more invasive. ROS production regulates the overexpression of TGF-β in cancer through both Smad and non-Smad signaling pathways that lead to EMT.

There is a possibility for potential therapeutic approaches for cancer that involve manipulating oxidative stress and the TGF-β pathway. Similar to vitamins C and E, several antioxidants might work as possible drugs to neutralize oxidative stress during cancer progression. The application of some antioxidants, such as N-acetylcysteine or Nox inhibitors, has proven to be effective for preventing cell proliferation and metastasis in cancer cells [101]. Correspondingly, the TGF-β pathway could be a point of focus for an anticancer pharmacological approach, specifically regarding the main mediator of TGF-β activities facilitating immunosuppression and stimulating angiogenesis, as well as EMT in cancer cells [4]. In addition, TGF-β signaling related to T-cell-specific blockade could increase antitumor immunity through the cytotoxic T-cell response [94].

### 5.5. Redox Modulation of TGF-β Signaling

Redox pathways control TGF-β/Smad signaling through feed-forward control, as irradiation or ion-catalyzed ROS formation provokes the transition of the latent form of TGF-β to an active form, representing a highly effective mechanism controlling the activation of TGF-β signaling [102]. Treatment with the most significant reactive aldehyde, 4-hydroxynonenal (4-HNE), increases TGF-β expression in cultured macrophages [103]. Furthermore, ROS may affect the regulation of TGF-β-generated Smad2/3 activation. Numerous antioxidant agents and Nox4 gene silencing considerably inhibit TGF-β-generated Smad2/3 phosphorylation in cardiac fibroblasts [104]. In fact, the Smad2 phosphorylation level is considerably reduced in Nox4-knockout bleomycin-induced pulmonary fibrosis mice [105]. Even though exogenous H_2_O_2_ is unsuccessful in regulating Smad2 phosphorylation, antioxidant treatment has been shown to successfully hinder the TGF-β-induced phosphorylation of Smad2 in kidney proximal tubular epithelial cells [71]. In cultured pulmonary fibroblasts, Smad2/3 phosphorylation by Nox4 modulation has also been observed [106]. Therefore, NADPH oxidase and ROS might play significant roles in enabling TGF-β-generated Smad2/3 activation.

In the GS domain, the phosphorylation of the type I receptor (ALK5) on serine and threonine residues is necessary for the initiation of TGF-β signaling. At the same time, phosphorylated ALK5 consecutively results in the phosphorylation of Smad2/3 in the C-terminal SXS motif and initiates downstream signaling [107]. Furthermore, redox-sensitive protein phosphatase 1 (PP1) and PP2A can be inactivated by ROS molecules such as H_2_O_2_, can dephosphorylate ALK5, and may lead to higher activation of ALK5 and phosphorylation of Smad [107,108]. ROS-induced oxidation and inactivation-sensitive PTEN [109], when in a phosphorylated state, may cause a reduction in Smad2/3 phosphorylation [107].

Smad-independent mechanisms can also regulate TGF-β signaling. TGF-β may initiate additional signaling pathways involving c-JNK and p38, which are redox sensitive, and, in the cytoplasm, they can be activated by ROS [109,110]. JNK and p38 may sequentially improve the transcriptional activities of Smad proteins through specific Smad3 phosphorylation or, secondarily, by fostering Smad3′s association with the transcriptional coactivator p300 [110].

## 6. Conclusions

TGF-β and ROS play significant roles in the progression of cancer. The processes facilitated by ROS come together with the initiation of TGF-β signaling and, thus, differentially impact early cancer development and metastatic spread. Furthermore, the ROS level affects both anti- and pro-cancer effects that subsequently vary depending on the situations of the cells and the stage of cancer. Consequently, these factors influence the potential for utilizing ROS as a therapeutic target.

**Figure 4:**
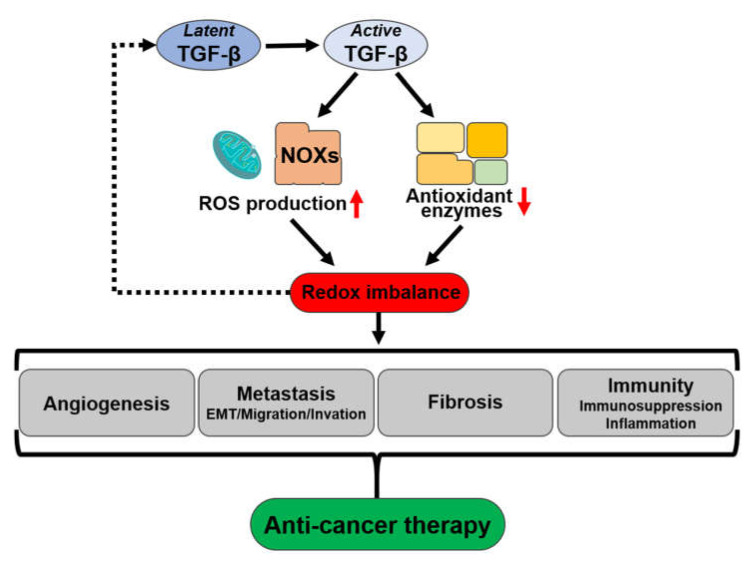
Crosstalk of TGF-β and oxidative stress in cancer and the therapeutic targets.

Crosstalk among oxidative stress/ROS and TGF-β occurs in cancer cells, i.e., TGF-β controls oxidative stress by boosting ROS production and controlling the antioxidant systems, whereas ROS control the TGF-β signaling pathway, particularly encouraging EMT, which promotes cancer invasiveness. Therefore, as the ROS levels are higher in cancer cells than normal cells, cancer cells might be more vulnerable than normal cells to an increase in ROS, providing a therapeutic opportunity (Figure 4).

Taken as a whole, this review endeavors to better elucidate the interaction between TGF-β and ROS. We think that the inhibition or control of the amplification loop between TGF-β and the ROS system in cancer cells could reduce tumor development and metastasis, weakening tumor dissemination, proliferation, and survival. Finally, explaining the complicated relationship and functions of TGF-β and oxidative stress in cancer is significant for identifying their involvement in initiation, development, and tumor metastasis and could ultimately reveal possible combinatory therapeutics for upcoming tests for cancer in humans.

## Data Availability

Not applicable.
